# Passivity-Based Control for Output Voltage Regulation in a Fuel Cell/Boost Converter System

**DOI:** 10.3390/mi14010187

**Published:** 2023-01-11

**Authors:** Carlo A. Beltrán, Luis H. Diaz-Saldierna, Diego Langarica-Cordoba, Panfilo R. Martinez-Rodriguez

**Affiliations:** 1 Faculty of Sciences, Autonomous University of San Luis Potosi (UASLP), Av. Chapultepec 1570, San Luis Potosi 78295, Mexico; 2 Control and Dynamical Systems Division, The Institute for Scientific and Technological Research of San Luis Potosi (IPICYT), Camino a la Presa de San Jose 2055, San Luis Potosi 78216, Mexico

**Keywords:** fuel cells, power converters, passivity-based control, parameter estimation

## Abstract

In this paper, a passivity-based control (PBC) scheme for output voltage regulation in a fuel-cell/boost converter system is designed and validated through real-time numerical results. The proposed control scheme is designed as a current-mode control (CMC) scheme with an outer loop (voltage) for voltage regulation and an inner loop (current) for current reference tracking. The inner loop’s design considers the Euler–Lagrange (E-L) formulation to implement a standard PBC and the outer loop is implemented through a standard PI controller. Furthermore, an adaptive law based on immersion and invariance (I&I) theory is designed to enhance the closed-loop system behavior through asymptotic approximation of uncertain parameters such as load and inductor parasitic resistance. The closed-loop system is tested under two scenarios using real-time simulations, where precision and robustness are shown with respect to variations in the fuel cell voltage, load, and output voltage reference.

## 1. Introduction

Interest in fuel cells (FCs) has been increasing because of the technology’s advantages such as high efficiency, zero $CO_{2}$ emissions, high power density, and scalability [1,2,3,4]. Additionally, FC technology is helping achieve an energy transition towards greener fuels. For instance, several countries are investing in the development of FC-related industries, such as green hydrogen production, FC-powered electrical vehicles (EVs), buses, trains, and large-scale stationary power generation systems [5]. Among FCs, the proton-exchange membrane fuel cell (PEMFC) stands out for its efficiency (up to 72%), low operating temperature, compactness, and applicability in low-, medium-, and high-power systems [6,7,8]. Note that the output voltage generated by a single FC is low (less than 1.2 V), so, in order to supply the desired demand series, parallel arrangements are created to form stacks. Additionally, auxiliary systems (such as valves, pumps, and air compressors) are required in order to obtain a PEMFC energy generation system (EGS).Compared to green EGSs such as photovoltaic systems or wind turbines, PEMFC EGSs are a non-intermittent energy source that converts chemical energy to electrical energy, heat, and water via an exothermic reaction that consumes hydrogen and oxygen [9]. However, drawbacks such as its strongly nonlinear characteristics [10], slow dynamic response [11], lifetime reduction due to the fuel starvation phenomena (voltage drop due to a rapid load demand) [12], and unregulated output voltage make FCs require additional systems in order to increase their reliability and durability [13,14].

With switching DC–DC converters as the interface between PEMFC EGS and an electrical load, it is possible to obtain a regulated output voltage, which usually is required to be higher than the input. The combination of a fuel cell EGS feeding an electrical load through a DC–DC converter as the interface is known as a *fuel cell system* [15], a widely studied topology. With respect to the DC–DC converter, a low-ripple input current is required, as a high ripple current has a negative effect on the PEMFC membrane, thus reducing its lifetime [16]. Additionally, high voltage gain and good transient response are desired [12]. To this end, there are two types of converters: isolated (galvanic isolation) and non-isolated. Although isolated converters achieve very high voltage gains and electrically isolate the power source from the load, due to drawbacks such as power losses, high cost, and larger size, non-isolated converters are preferred [17]. Thus, a well-known step-up converter is the traditional boost converter, with a non-pulsing input current advantage. Although theoretically, its voltage gain can be as high as ten, it is less than that due to parasitic elements. Moreover, at high gains, the efficiency of the converter is reduced [18]. Thus, in order to achieve high gain converters, the use of coupled inductors, switched capacitor charge pumps, and voltage multipliers was reported in [17]. Moreover, [19] reported a floating interleaved boost converter with the use of coupled inductors. Due to their non-pulsating input currents, these converters are suitable for use in fuel cell applications. However, these topologies require more components, are more complex to control, and require more computational power. Therefore, the boost converter has attracted much attention in fuel cell and controller design applications.

The non-minimum phase (NMP) behavior and bi-linear characteristics are part of the dynamics of the DC–DC boost converter. The NMP occurs in the transfer function of the duty cycle to the output voltage. Therefore, a direct approach to output voltage regulation results in unstable behavior, thus increasing the difficulty of this task [20]. To address this issue, voltage regulation is achieved indirectly through inductor current regulation. In this way, current-mode control (CMC) provides a method for regulating the output voltage. This is a multi-loop scheme with an outer loop (voltage) for voltage regulation through current reference generation, and an inner loop (current) for current reference tracking through duty cycle generation. This scheme has features such as fast transient response, improved multi-loop stability, and direct control over the current (mandatory for industrial applications) [6,12]. The use of nonlinear controllers to deal with control problems related to DC–DC converters has drawn considerable interest, including nonlinear control strategies such as sliding mode control (SMC) [20,21,22], fuzzy logic control (FLC) [23], differential flatness [24], backstepping [25,26], and passivity-based control (PBC) [6,27,28]. Among them, PBC has attracted a great deal of attention in power converters for its physical-related concepts such as damping, interconnection, and energy. In this methodology, the passivity property is put forward, thus, a system is said to be passive when it cannot store more energy than is supplied to it from the outside, with the difference being the dissipated energy [29]. Its objective is to render a system passive with respect to the desired energy storage function that has a minimum at the desired equilibrium point [30]. Although there are several branches, PBC is mainly classified into two groups: standard PBC, and interconnection and damping assignment passivity-based control (IDA-PBC), where standard PBC deals with Euler–Lagrange (E-L) systems and IDA-PBC with port-controlled Hamiltonian (PCH) systems [31]. Note that the standard PBC is selected for this work. Thus, this methodology is performed with two steps: *damping injection* to the system to ensure asymptotic stability and *energy shaping* to obtain a single global minimum at the desired equilibrium.

In the literature, solving the output voltage regulation control problem has been addressed by multiple nonlinear control strategies. In [25], for example, an adaptive multi-loop controller for output voltage regulation for a PEMFC boost fuel cell system was reported. The outer loop was designed with a PI action and the inner loop was designed with backstepping. Additionally, immersion and invariance (I&I) theory approach was used to estimate the load. It was validated via experimentation, and its major drawback was found to be a computationally demanding control law. In [22], a multi-loop controller for an interleaved boost fuel cell system was reported. The outer loop was designed with active disturbance rejection control (ADRC) and the inner loop was designed with super-twisting SMC. The ADRC output loop increases the robustness of the overall system by addressing the uncertainties in the load and input voltage. It was validated via experimentation and its major drawback was found to be the chattering phenomenon of SMC, which could lead to high-frequency vibration of the system. In [28], adaptive single-loop control for output voltage regulation for a Cùk converter was addressed. PI-PBC was used to obtain the control law, resulting in an expression that only requires knowledge of the load and states, and thus parameter uncertainty does not affect the controller performance. To estimate the load and increase the robustness of the control law, the I&I theory approach was used. For this study, stability was assured via PCH systems, and controller performance was validated through numerical simulations. In [32], a PEMFC boost fuel cell system was controlled for output power regulation dealing with the auxiliary units of the EGS (air compressor, air cooler, and water-based heat exchange, among others). The controller was designed with SMC and two nonlinear observers were designed with an extended Kalman filter and a sliding mode observer. Note that this resulted in a high-order system, thus, validation of its performance requires high-cost systems (experimentally) and is computationally demanding (real-time simulations).

The contribution of this paper relies on the design of an adaptive CMC scheme to solve the output voltage regulation for a PEMFC EGS supplying a boost converter. An outer (voltage) loop was designed to generate the desired current reference with a PI action over the voltage error. Then, an inner (current) loop was designed with PBC to take advantage of the passive map of the control signal to the inductor current [29]. Moreover, aiming to deal with the issue of unknown but constant parameters and to increase the robustness of the inner loop controller, an adaptation law based on I&I theory, detailed in [33,34], was designed to estimate both the parasite resistance and the load. The overall scheme resulted in a nonlinear adaptive multi-loop controller, which ensures asymptotic convergence of all error signals to the origin via Lyapunov stability. Additionally, by doing real-time numerical simulation, it is possible to evaluate the proposed controller’s computational feasibility and performance. Real-time simulation results have shown precise output voltage regulation through proper current tracking despite variations in the input voltage and sudden stepwise changes in the load and output voltage reference.

Among others, the main contributions of this work in relation to output voltage regulation of the boost converter are as follows:

- The design of a current-mode adaptive multi-loop PBC scheme for output voltage regulation of a fuel cell system.
- The design of an adaptive law based on I&I for inductor parasitic and load resistances.
- To the best of the authors’ knowledge, there are no studies of PBC applied to the fuel cell boost converter system reported in the literature.

The remainder of this work is organized as follows. In Section 2, several PEMFC static models are compared, and a dynamical model considering the boost converter is detailed. Afterward, the proposed adaptive multi-loop control scheme is presented in Section 3. Real-time simulation scenarios are provided in Section 4, and some final concluding remarks are presented in Section 5.

## 2. System Description

**Figure 1:**
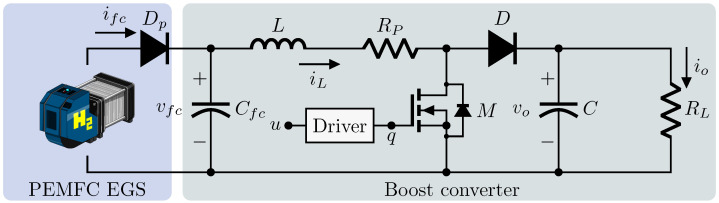
Fuel cell system composed of a PEMFC EGS feeding a purely resistive load through a DC–DC boost converter as the interface.

This section describes the analysis, physical assumptions, and methods to obtain a representation of the system under study. As depicted in Figure 1, the fuel cell system is formed by a PEMFC EGS feeding a purely resistive load through a DC–DC boost converter as the interface. The components are an EGS, a fully automated fuel cell stack with a rated output power of about 1.2 kW at 24 V; the diode $D_{p}$, that prevents reverse current flow to the PEMFC; the coupling capacitor $C_{fc}$, the input inductor *L*; the MOSFET transistor *M*; the output capacitor *C*; the load $R_{L}$, which is considered purely resistive; and the parasitic resistance $R_{p}$ of the inductance that is taken into account to increase the accuracy of the model. The currents $i_{fc}$, $i_{L}$, and $i_{o}$ are the fuel cell, inductor, and output average currents, respectively. The voltages $v_{fc}$ and $v_{o}$ are the PEMFC and converter output voltage, respectively, and *u* is the duty cycle to generate the PWM trigger signal *q*.

### 2.1. Fuel Cell Models Based on Experimental Data

A PEMFC EGS is integrated by several subsystems such as a PEMFC stack, a hydrogen supply system that regulates the hydrogen flow or pressure through a valve, an air supply system designed to increase the power density, a humidification system to prevent dehydration of the FC membrane, and a cooling system to reduce the temperature of the inlet air [11]. In this way, PEMFCs are ultimately electrochemical devices that convert chemical energy to electrical energy via an exothermic reaction that consumes hydrogen and oxygen [9]. The PEMFC is internally composed of an electrolyte, an anode (negative electrode), and a cathode (positive electrode). In order to produce electricity, two electrochemical reactions are performed. The hydrogen is separated at the anode into positive ions ($H^{+}$), negative ions ($e^{-}$), and heat; then, these are filtered with a membrane. Because the membrane is not permeable to electrons, they are collected in an external circuit to produce electricity. Afterward, the positive ions flow and react with oxygen at the cathode to produce water [1]. A widespread way of classifying fuel cell types is by the electrolytic membrane [2]. In this way, there exists alkaline FC, PEMFCs, molten carbonate FCs, and direct carbon FCs, among others. Note that some FCs operate at high temperatures (greater than 900 °C) and others have low efficiency (less than 15%). The PEMFC is selected for this work because it stands out for its high efficiency (up to 72%), low operating temperature, compactness, and applications in low-, medium-, and high-powered systems [6,7,8]. However, the major drawbacks of FC technology are its high cost, low durability, and unregulated output voltage [3]. Note that its resulting dynamics are the interaction of multiple physical and complex subsystems; therefore, to simplify the analysis, only the dynamics of the PEMFC voltage and current are considered.

**Figure 2:**
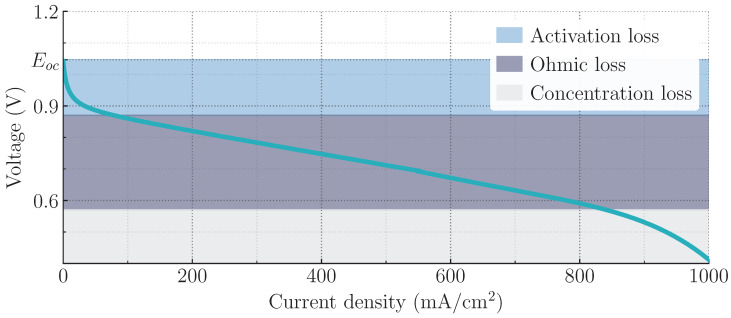
Typical relationship between the output voltage $v_{cell}$ and current density for a fuel cell working at low temperature and air pressure.

Particularly, the output voltage generated by a single fuel cell is approximately $v_{cell}$ = 1.2 V, which is the result of four different voltage phenomena [35], this is:

(1) $$v_{cell}=E_{oc}-v_{act}-v_{ohm}-v_{con},$$

where $E_{oc}$ is the open-circuit voltage (also called reversible or Nernst voltage), $v_{act}$ is the activation voltage loss that represents the slowness of the electrode surface reactions, $v_{ohm}$ is the ohmic voltage loss that represents the resistance to the flow of electrons and ions, and $v_{con}$ is the voltage concentration loss that represents the reduction in the concentration of the reactants at the surface of the electrodes [1,4,36]. For instance, in Figure 2, the relationship between the output voltage $v_{cell}$ and current density is shown. Note that $v_{cell}$ decays (in a nonlinear manner) below the $E_{oc}$ when the demanded current density increases.

The development of precise and easy-to-use dynamical models for fuel cell stacks is a subject of current interest due to its complex dynamics that result from the interaction of multiple physical phenomena [14]. For example, in [35], the internal dynamics of a PEMFC are modeled with the use of three electrical equivalent circuits to capture complex electric, pneumatic, and thermal dynamics, which yields a high-order nonlinear system that is difficult to implement in simulations considering additional power converters dynamics. Moreover, in [11], a ninth-order model was presented. The dynamics of transitory phenomena and auxiliary elements of the PEMFC stack were contemplated, such as the dynamics of the compressor, the vapor masses in the cathode and anode, and pressure of gasses, among others. Note that due to its complexity, it is not suitable for numerical analysis in applications where high-frequency power electronics systems are used to process the PEMFC EGS energy. On the other hand, several static models based on experimental data with applications in power conversion have been reported in the literature. For example, in [37], a PEMFC semiempirical model that only requires three parameters and is continuous for a wide range of currents was presented. Particularly, this rational model is described by the fuel cell voltage–current relationship:

(2) $$v_{fc}\left(i_{fc}\right)=\frac{E_{oc}}{1+{\left(\frac{i_{fc}}{I_{h}}\right)}^{\gamma }},$$

where the parameters γ and $I_{h}$ were experimentally obtained through root mean square approximation for a different relative humidity of inlet air. Another case is the fifth-degree polynomial model presented in [6]:

(3) $$v_{fc}\left(i_{fc}\right)=E_{oc}+c_{1}i_{fc}+c_{2}i_{fc}^{2}+c_{3}i_{fc}^{3}+c_{4}i_{fc}^{4}+c_{5}i_{fc}^{5},$$

where the polynomial coefficients $c_{1}$ to $c_{5}$ are obtained by fitting the experimental data of the stack. Additionally, in [25], a two-termed nonlinear power function model is presented. It uses three parameters that are also computed using experimental data. The relationship between the voltage and current in this case is described by:

(4) $$v_{fc}\left(i_{fc}\right)=E_{oc}-ai_{fc}^{b},$$

where the parameters *a* and *b* are obtained from experimental data approximation. A more complex and accurate model is presented in [38]. In an attempt to capture the nonlinear dynamics of the PEMFC, this model requires multiple parameters that depend on the PEMFC stack characteristics. As discussed previously, the nonlinear dynamics can be represented by the major voltage losses, i.e., activation, ohmic, and concentration losses. In this way, several PEMFC parameters are used to implement the Larminie and Dicks model given by:

(5) $$\begin{array}{cc} v_{fc}\left(i_{fc}\right)= & E_{oc}-A\cdot \log\left(\frac{i_{fc}+i_{n}}{i_{0}}\right)-R_{m}\left(i_{fc}+i_{n}\right)+B\cdot \log\left(1-\frac{i_{fc}+i_{n}}{i_{lim}}\right), \end{array}$$

where $i_{0}$ is the exchange current, *A* is the slope of the Tafel line, $i_{lim}$ is the current limit, *B* is a mass transfer constant, $i_{n}$ is the internal current, and $R_{m}$ is the membrane resistance. The second term of (5) represents the activation voltage loss with the Tafel equation which considers the voltage loss as the potential difference between the measuring electrode and its theoretical equilibrium point. The third term represents the voltage loss due to the electrical resistance of the electrolyte and electrodes with Ohm’s law. The last term is used to estimate the concentration voltage drop due to the pressure decrease caused by the consumption of fuel and oxygen [1].

**Figure 3:**
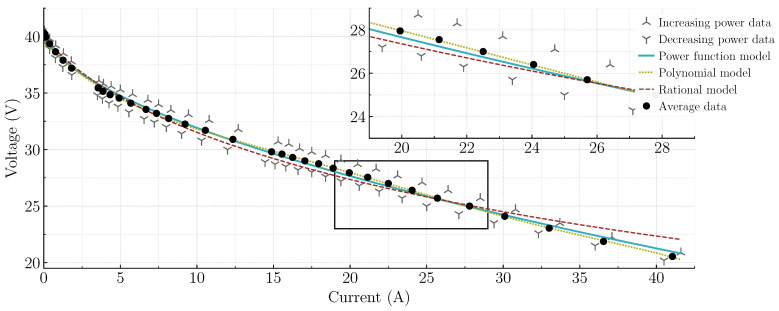
Models and experimental data comparison to represent the polarization curve of the PEMFC EGS. Experimental data are obtained by increasing/decreasing the power demand.

As outlined in the system description, in this study the EGS considers a Nexa fully automated fuel cell stack with a rated output power of about 1.2 kW. With the intention of modeling the static behavior of this EGS, experimental data were collected. As illustrated in Figure 3, the experimental data were obtained by increasing the output power from the open circuit operation (0 A, 40.5 V) to about 70% of the nominal power (41.6 A, 20.89 V). Furthermore, in order to capture the hysteresis phenomenon, the output power was decreased until open circuit operation was reached. Because the hysteresis phenomenon is a complex nonlinear behavior that is difficult to model, averaged experimental data are used instead. In this way, the model in [37], the fifth-degree polynomial, and the two-termed nonlinear power function models described above are computed from the averaged experimental data of this particular PEMFC EGS. It is important to mention that a logarithmic transformation and a linear regression on the average data are performed to obtain the parameters required by each model [26]. As can be seen in Figure 3, the most accurate model for representing the three major voltage losses is the fifth-degree polynomial model. On the other hand, the least accurate is the model in [37], as can be seen after the activation voltage loss. Therefore, the two-termed nonlinear power function is a good choice because it only requires three parameters, it is less computationally demanding than the polynomial, and is more accurate than the model in [37]; therefore, it is the one selected in this work to represent the PEMFC voltage–current relationship.

### 2.2. Fuel-Cell Coupled to the Boost Converter Model

This section is devoted to modeling the DC–DC converter with a purely resistive load and input voltage supplied by the PEMFC. To simplify the obtainment of its dynamics and consequently the controller design, the average model (ripple-free) for the converter is analyzed. Additionally, the following assumptions are made:

**Assumption 1**. *The boost converter is operating under continuous conduction mode (CCM), i.e., the current $i_{L}$ is always positive. Its operation in discontinuous conduction mode is not considered for this study*.

**Assumption 2**. *The power switches M, $D_{p}$, and D are considered ideal*.

**Assumption 3**. *
The passive elements are considered ideal (time invariant, frequency independent, and lossless).*

**Assumption 4**. *
The state variables are measurable (inductor current and capacitors voltages).*

Applying Kirchhoff’s current and voltage laws the Fuel cell coupled to the boost converter is modeled as a third-order nonlinear state-space model:

(6) $$\begin{array}{cc} \frac{dv_{fc}}{dt} & =\frac{1}{C_{fc}}\left(i_{fc}\left(v_{fc}\right)-i_{L}\right),\\ \frac{di_{L}}{dt} & =\frac{1}{L}\left(v_{fc}-R_{p}i_{L}-\left(1-u\right)v_{o}\right),\\ \frac{dv_{o}}{dt} & =\frac{1}{C}\left(\left(1-u\right)i_{L}-\frac{1}{R_{L}}v_{o}\right), \end{array}$$

where the state variables are the coupling capacitor voltage $v_{fc}$, inductor current $i_{L}$, and output voltage $v_{o}$. Considering (4), the fuel cell current is:

(7) $$i_{fc}\left(v_{fc}\right)={\left(\frac{E_{oc}-v_{fc}}{a}\right)}^{\frac{1}{b}}.$$

Note that due to physical constraints and assumptions, the state variables and control signal are bounded according to:

(8) $$\begin{array}{cc} \; & v_{fc}\in E≜\left(v_{fc_{min}},E_{oc}\right)\subset R_{+},\\ \; & i_{L}\in I≜\left(i_{L_{min}},i_{L_{max}}\right)\subset R_{+},\\ \; & v_{o}\in V≜\left(v_{o_{min}},v_{o_{max}}\right)\subset R_{+},\;\\ \; & u\in U≜\left(0,u_{max}\right)\subset R_{+},\;u_{max}<1 \end{array}$$

where $R_{+}$ denote the positive real numbers. Due to CCM operation and safety reasons, the inductor current is bounded, the output voltage is bounded by the minimum PEMFC voltage and maximum output voltage, and because the control signal represents the duty cycle values greater than one is unfeasible, consequently, this signal is bounded by $u_{max}<1$. It is important to note that it is not appropriate to operate the converter with a control signal above this level. Hence, a saturation function is used to ensure that u∈U for all time.

Consider a steady state, that is, when the control signal remains constant $u=\bar{u}$, and the overall system (6) states do not change. The equilibrium points are obtained as:

(9) $$\begin{array}{cc} \; & {\bar{v}}_{fc}=E_{oc}-a{\left({\bar{i}}_{L}\right)}^{b},\\ \; & {\bar{i}}_{L}=\frac{{\bar{v}}_{fc}}{R_{L}{\left(1-\bar{u}\right)}^{2}+R_{p}},\\ \; & {\bar{v}}_{o}=\frac{{\bar{v}}_{fc}R_{L}\left(1-\bar{u}\right)}{R_{L}{\left(1-\bar{u}\right)}^{2}+R_{p}}. \end{array}$$

Observe that the computation of the equilibrium points (9) requires the knowledge of the unknown parameters $R_{p}$ and $R_{L}$. Additionally, there exists an algebraic loop between ${\bar{v}}_{fc}$ and ${\bar{i}}_{L}$; therefore, (9) cannot be used as a reference for the controller design stage.

**Remark 1**. *In a real-life implementation, other parasitic resistances such as the diode and switch ON resistances, fuel-cell, output capacitor series, and leakage resistances are involved; however, in this work, we focus on the parasitic resistance $R_{p}$ because it is greater than the other parasitic resistances and produces major steady-state errors if it is not considered. For example, in [39], an experimental validation for output voltage regulation with an adaptive law that estimates both the uncertainty based on $R_{p}$ and on the load is detailed, and the results support this claim*.

### 2.3. Control Objectives

Consider that the fuel cell system (6) fulfills the Assumptions 1–4, and the control error variable is defined as:

(10) $$\tilde{x}≜x-x_{🟉},$$

where $x={\left[x_{1}\;\;x_{2}\;\;x_{3}\right]}^{⊤}={\left[v_{fc}\;\;i_{L}\;\;v_{o}\right]}^{⊤}$ is the state vector and $x_{🟉}={\left[x_{1🟉}\;\;x_{2🟉}\;\;x_{3🟉}\right]}^{⊤}$ the desired state vector to be designed. The following control objectives can be stated, namely current tracking and voltage regulation objectives:

O1. **Current tracking.** To fulfill this objective, an inner (current) control loop is designed. This loop assures the correct tracking of the state *x* towards the desired state $x_{🟉}$. Formally, this control objective is described as:
  (11) $$\lim_{t\to \infty }x\left(t\right)=x_{🟉}\left(t\right).$$
O2. **Voltage regulation.** To fulfill this objective, an outer (voltage) loop is designed to regulate the voltage $x_{2}$ at a constant reference voltage $v_{ref}\in V$. Formally, this control objective is described as:
  (12) $$\lim_{t\to \infty }x_{3}\left(t\right)=v_{ref}.$$

## 3. Passivity-Based Controller Design

This section presents the detailed obtainment of the solution to the output voltage regulation problem using standard PBC and the E-L formalism. To begin, a system is passive when it cannot store more energy than is supplied to it from the outside, with the difference being the dissipated energy [29]. The PBC approach is a widely recognized control methodology with a particular characteristic that respects and exploits the nonlinear structure of the system. To assure system stability, this methodology aims to render it passive with respect to the desired storage function that has a minimum at the desired equilibrium point [30]. Based on these ideas, the controller is designed to regulate the output voltage indirectly (due to the NMP behavior of the output voltage to the control signal) by controlling the inductor current directly. For this purpose, two control loops are designed: an outer (voltage) loop to generate the desired current reference with a PI action over the voltage error and an inner (current) loop designed with PBC to take advantage of the passive map $u\to x_{1}$[29]. Furthermore, to address the issue that the load and parasitic resistance are unknown but constant, an adaptive law based on I&I theory is designed [34]. The idea is to compute the estimation ${\hat{\theta }}_{L}$ of the load conductance $\theta_{L}=1/R_{L}$ and the estimation ${\hat{R}}_{p}$ of the parasite resistance $R_{p}$ and use these estimations in the inner loop controller in a certainty-equivalent manner. Note that this theory’s applications have been widely reported for parameter estimators, stabilizing laws, and state observers for nonlinear systems. Consequently, the controller results in an adaptive multi-loop scheme, which is summarized in the following sections.

### 3.1. Outer Control Loop Design

Because the equilibrium point for the inductor current in (9) cannot be used as a reference, the outer loop is constructed with a PI control over the output voltage error to compute the appropriate current reference for the PBC method. Hence, the error for this loop is defined as the difference between the constant voltage reference $v_{ref}$ and output voltage as:

(13) $$e≜v_{ref}-x_{3},$$

then, according to [40,41,42,43], the desired current reference can be computed as:

(14) $$x_{2🟉}=k_{p}e+k_{i}\int_{0}^{t}e\left(\tau \right)\;d\tau ,$$

where $x_{2🟉}\in I$ and $k_{p},k_{i}>0$, being the tuning control gains to be selected. Recalling that the controller is designed to ensure that the control objectives are met, i.e., $x=x_{🟉}$ and $x_{3}=v_{ref}$. Consequently, an inner loop is required to compute the control signal *u* for output voltage regulation through current reference tracking.

### 3.2. Inner Control Loop Design

In this passivity-based methodology, the steps to compute the control law are as follows: the system is transformed into an E-L system, an energy function for the closed loop is designed, then *damping injection* is performed to modify the dissipation function, and ensure asymptotic stability, and finally, *energy-shaping* to obtain a single global minimum at the desired equilibrium point [30]. Thus, the fuel cell system (6) is transformed into an E-L system:

(15) $$D\dot{x}-J\left(u\right)x+Rx=E,$$

where D denote the generalized inertia matrix, J the interconnection matrix with the skew-symmetric property ($J\left(u\right)=-J{\left(u\right)}^{⊤}$), R the dissipation matrix with the diagonal and positive semi-definite properties ($R=R^{⊤}\geq 0$), and E the external sources vector, with the matrices and vector:

(16) $$D=\left[\begin{array}{ccc} C_{fc} & 0 & 0\\ 0 & L & 0\\ 0 & 0 & C \end{array}\right],\;J(u)=\left[\begin{array}{ccc} 0 & -1 & 0\\ 1 & 0 & -(1-u)\\ 0 & \left(1-u\right) & 0 \end{array}\right],\;R=\left[\begin{array}{ccc} 0 & 0 & 0\\ 0 & R_{p} & 0\\ 0 & 0 & \theta_{L} \end{array}\right],E=\left[\begin{array}{c} i_{fc}\\ 0\\ 0 \end{array}\right],$$

where $\theta_{L}=1/R_{L}$ is the conductance of the load. For additional information regarding E-L systems, the reader may refer to [29], where these parameters are selected as:

As discussed previously, the next step is the design of an energy function for the closed-loop system:

(17) $$H\left(\tilde{x}\right)=\frac{1}{2}{\tilde{x}}^{⊤}D\tilde{x},$$

where $\tilde{x}$ is the inner loop control error defined in (10). Recalling that $x=\tilde{x}+x_{🟉}$, the dynamics in (15) can be written as:

(18) $$D\left(\dot{\tilde{x}}+{\dot{x}}_{🟉}\right)-J\left(u\right)\left(\tilde{x}+x_{🟉}\right)+R\left(\tilde{x}+x_{🟉}\right)=E.$$

With the intention of modifying the closed-loop system dissipation, the *damping injection* stage is performed. With this objective, the desired dissipation is designed by:

(19) $$R_{d}\tilde{x}=\left(R+R_{i}\right)\tilde{x},$$

where $R_{i}=\mathrm{diag}\left\{r_{1},\;\;r_{2},\;\;r_{3}\right\}>0$ is the injected damping matrix with $r_{1},\;r_{2},\;r_{3}>0$, being the tuning control gains to be selected. Recall that these gains can be physically described as *virtual resistances* (damping) injected via feedback to the system. Therefore, the term $R_{i}\tilde{x}$ is added in both sides of (18), and after some computations, the resulting control error dynamics can be expressed as:

(20) $$D\dot{\tilde{x}}-J\left(u\right)\tilde{x}+R_{d}\tilde{x}=\Psi ,$$

where Ψ is a perturbation term defined as:

(21) $$\Psi ≜E-\left(D{\dot{x}}_{🟉}-J\left(u\right)x_{🟉}+Rx_{🟉}\right)+R_{i}\tilde{x}.$$

The next stage in the controller design is the *energy shaping* of the error dynamics (20) to ensure stability. It is important to mention that the closed-loop energy function (17) is a candidate Lyapunov function, i.e., H(0)=0, and $H(\tilde{x})>0$ when $\tilde{x}\neq 0$. Therefore, aiming to prove Lyapunov stability, the time derivative of the closed-loop energy function $H(\tilde{x})$ in (17), along the trajectories of the error dynamics (20), is computed as:

(22) $$\dot{H}\left(\tilde{x}\right)={\tilde{x}}^{⊤}D\dot{\tilde{x}}={\tilde{x}}^{⊤}\left(J\left(u\right)-R_{d}\right)\tilde{x}+{\tilde{x}}^{⊤}\Psi ,$$

and because J(u) is skew-symmetric, satisfies with ${\tilde{x}}^{⊤}J\left(u\right)\tilde{x}=0$, therefore:

(23) $$\dot{H}\left(\tilde{x}\right)=-{\tilde{x}}^{⊤}R_{d}\tilde{x}+{\tilde{x}}^{⊤}\Psi .$$

Note that if the perturbation term (21) remains zero for all time points (*energy shaping*), the time derivative of the energy function results in:

(24) $$\dot{H}\left(\tilde{x}\right)=-{\tilde{x}}^{⊤}R_{d}\tilde{x}<0,$$

a negative definite function, and consequently the origin of (20) is asymptotically stable, thus $x\to x_{🟉}$ as t→∞ [44].

The previous stability analysis is based on the assumption that Ψ=0 for all time, which implies that:

(25) $$E=D{\dot{x}}_{🟉}-J\left(u\right)x_{🟉}+Rx_{🟉}-R_{i}\tilde{x},$$

is required to be satisfied for all time as well. Because the desired current $x_{2🟉}$ is already defined in (14), the only two remaining signals to define to fulfill (25) are *u*, $x_{1🟉}$ and $x_{3🟉}$; therefore, if *u* and the controller auxiliary dynamics ${\dot{x}}_{1🟉}$ and ${\dot{x}}_{3🟉}$ are selected as

(26) $$\begin{array}{cc} \; & u=1-\frac{1}{x_{3🟉}}\left(x_{1🟉}-L{\dot{x}}_{2🟉}-R_{p}x_{2🟉}+r_{2}{\tilde{x}}_{2}\right), \end{array}$$

(27) $$\begin{array}{cc} \; & {\dot{x}}_{1🟉}=\frac{1}{C_{fc}}\left(i_{fc}-x_{2🟉}+r_{1}{\tilde{x}}_{1}\right), \end{array}$$

(28) $$\begin{array}{cc} \; & {\dot{x}}_{3🟉}=\frac{1}{C}\left(\left(1-u\right)x_{2🟉}-\theta_{L}x_{3🟉}+r_{3}{\tilde{x}}_{3}\right), \end{array}$$

then, Ψ=0∀t≥0. It is important to mention that the computation of Equations (26)–(28) requires the unknown parameters $R_{p}$ and $\theta_{L}$ and the time derivative of the current reference ${\dot{x}}_{2🟉}$. By designing an adaptive law based on I&I theory, it is possible to estimate these unknown parameters, and the remaining signal ${\dot{x}}_{2🟉}$ is obtained from (14) as:

(29) $${\dot{x}}_{2🟉}=k_{p}\dot{e}+k_{i}e.$$

As a limitation of this control scheme, observe that the last expression requires the term $\dot{e}$, which in turn requires ${\dot{x}}_{2}$, a function of the control signal *u*; therefore an algebraic loop in (26) is identified. After solving this issue, the control signal can be rewritten as:

(30) $$\begin{array}{c} u=1-\frac{1}{Cx_{3🟉}-k_{p}Lx_{2}}\left(C\left[x_{1🟉}+r_{2}{\tilde{x}}_{2}-R_{p}x_{2🟉}-k_{i}Le\right]-k_{p}L\theta_{L}x_{3}\right), \end{array}$$

where, to avoid a singularity ($Cx_{3🟉}-k_{p}Lx_{2}=0$), the proportional gain must accomplish:

(31) $$k_{p}\neq \frac{Cx_{3🟉}}{Lx_{2}},$$

in this way, the condition (31) holds if $k_{p}$ is chosen according to the relation:

(32) $$k_{p}\notin \left[\frac{Cv_{o_{min}}}{Li_{L_{max}}},\frac{Cv_{o_{max}}}{Li_{L_{min}}}\right].$$

It should be noted that this constraint on the proportional gain is dependent on knowledge of known system parameters as well as the lower and upper bounds of the output voltage and inductor current, respectively, acquired from physical considerations.

### 3.3. Adaptive Law Design

With the goal of handling the issue of unknown but constant parameters and to increase the robustness of the proposed controller scheme (30), an adaptation law based on I&I theory detailed in [33,34] is designed. It is well documented in the literature that this theory has been extensively applied to the design of nonlinear systems, state observers [45], stabilizing control laws [46], and parameter estimators [47]. The idea in this work is to compute both the estimation ${\hat{\theta }}_{L}$ of the load conductance $\theta_{L}=1/R_{L}$ and ${\hat{R}}_{p}$ of the parasite resistance $R_{p}$ and use these values in a certainty-equivalent way for the inner loop controller. In this way, according to the I&I theory, the first stage is to define the estimation error as:

(33) $$\tilde{\delta }≜\delta -\hat{\delta },$$

where $\delta ={\left[R_{p},\;\;\theta_{L}\right]}^{⊤}\in R_{+}^{2}$ is the vector of the unknown parameters (which elements are the dissipation matrix parameters in (16)) and $\hat{\delta }={\left[{\hat{R}}_{p},\;{\hat{\theta }}_{L}\right]}^{⊤}\in R_{+}^{2}$, is the vector of estimated parameters. For the next stage, according to the I&I theory, the estimation vector is defined as the sum of an integral term and a proportional term:

(34) $$\hat{\delta }=\xi +\eta \left(x_{c}\right),$$

where $\xi ={\left[\xi_{1},\xi_{2}\right]}^{⊤}\in R^{2}$ is the integral term, $\eta \left(x_{c}\right)={\left[\eta_{1},\eta_{2}\right]}^{⊤}\in R^{2}$ is the proportional term, and $x_{c}={\left[x_{2},x_{3}\right]}^{⊤}$ is the state partition corresponding to converter states: the inductor current and output voltage. Recall that $R_{p}$ and $\theta_{L}$ in (6) only affect $x_{2}$ and $x_{3}$ dynamics, respectively. Consequently, the dynamics of (33) by assuming unknown but constant parameters, i.e., $\dot{\delta }=0$, can be expressed as:

(35) $$\dot{\tilde{\delta }}=-\dot{\xi }-\frac{\partial \eta }{\partial x_{c}}{\dot{x}}_{c}.$$

Thus, the integral and proportional terms are designed to ensure $\tilde{\delta }\to 0$ as t→∞ and consequently $\hat{\delta }=\delta$. If the dynamics in (6) are considered, then the error dynamics in (35) can be rewritten as:

(36) $$\dot{\tilde{\delta }}=-\dot{\xi }-\frac{\partial \eta }{\partial x_{c}}D_{c}^{-1}\left[\left(1-u\right)J_{c}x_{c}-Y\left(x_{c}\right)\delta +E_{c}\right],$$

where $D_{c}=\mathrm{diag}\left\{L,C\right\}$, $Y\left(x_{c}\right)=\mathrm{diag}\left\{x_{2},x_{3}\right\}$, $E_{c}={\left[x_{1},0\right]}^{⊤}$ and

(37) $$J_{c}=\left[\begin{array}{cc} 0 & -1\\ 1 & 0 \end{array}\right].$$

Furthermore, because $\delta =\tilde{\delta }+\hat{\delta }$, then the estimation error dynamics results in

(38) $$\dot{\tilde{\delta }}=-\Lambda Y\left(x_{c}\right)\tilde{\delta },$$

if its internal dynamics and proportional term are selected as:

(39) $$\begin{array}{cc} \; & \dot{\xi }=\Lambda \left(E_{c}+\left(1-u\right)J_{c}x_{c}-Y\left(x_{c}\right)\hat{\delta }\right),\\ \; & \frac{\partial \eta }{\partial x_{c}}=-\Lambda D_{c}, \end{array}$$

where $\Lambda =\mathrm{diag}\{\lambda_{1},\;\lambda_{2}\}>0$ is a positive definite matrix with $\lambda_{1},\;\lambda_{2}>0$, being the tuning adaptation law gains to be selected. With the aim to prove convergence of the estimation error $\tilde{\delta }$ to the origin, a candidate Lyapunov function $V:R^{2}\to R$ is defined as:

(40) $$V\left(\tilde{\delta }\right)=\frac{1}{2}{\tilde{\delta }}^{⊤}\tilde{\delta },$$

then, the time derivative of (40) along the estimation error trajectory is computed as:

(41) $$\dot{V}\left(\tilde{\delta }\right)={\tilde{\delta }}^{⊤}\dot{\tilde{\delta }}=-{\tilde{\delta }}^{⊤}\Lambda Y\left(x_{c}\right)\tilde{\delta }.$$

**Figure 4:**
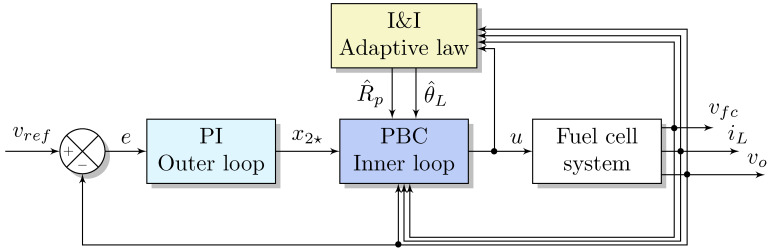
Block diagram of the E-L-PBC-based adaptive multi-loop controller.

Because Λ and $Y(x_{c})$ are positive definite matrices, (41) is always negative; therefore, it is assured that $\tilde{\delta }$ vanishes at the origin when t→∞, which indicates that $\hat{\delta }=\delta$. As previously outlined, the estimation is performed by the sum of the integral and the proportional terms to generate the vector of estimated parameters $\hat{\delta }$. So, the direct computation of the components of $\hat{\delta }$, considering (34) and (39), is:

(42) $$\begin{array}{cc} \; & {\hat{R}}_{p}=\lambda_{1}\left(\int_{0}^{t}\left[x_{1}-\left(1-u\right)x_{3}-{\hat{R}}_{p}x_{2}\right]d\tau -Lx_{2}\right),\\ \; & {\hat{\theta }}_{L}=\lambda_{2}\left(\int_{0}^{t}\left[\left(1-u\right)x_{2}-{\hat{\theta }}_{L}x_{3}\right]d\tau -Cx_{3}\right), \end{array}$$

which are used in the controller (30), to substitute the unknown parameters $R_{P}$ and $\theta_{L}$. Finally, the resulting passivity-based adaptive multi-loop controller is summarized in the following Proposition 1 and illustrated with the block diagram in Figure 4.

**Proposition 1**. *Consider the fuel cell system (6) verifying Assumptions 1–4 and the physical restrictions in (8). The dynamic nonlinear control law (30), with $x_{2🟉}$ computed by (14), $x_{1🟉}\in E$ by (27), $x_{3🟉}\in V$ by (28), and the adaptation law (42) with tuning gains $r_{1},\;r_{2},\;r_{3},k_{i}\;,k_{p},\lambda_{1}\;,\lambda_{2}\in R_{+}$ sufficiently large, and an initial condition $x_{1🟉}\left(0\right)\in E$, $x_{3🟉}\left(0\right)\in V$, is well-defined and asymptotically stabilizes the system trajectories towards the desired equilibrium point $\left({\bar{v}}_{fc},{\bar{i}}_{L},{\bar{v}}_{o},{\bar{x}}_{1🟉},{\bar{x}}_{3🟉}\right)\in E^{2}\times I\times V^{2}$*.

**Remark 2**. *The overall control scheme proposed in this work assures the control objectives; however, it presents the following two drawbacks: a non-conservative restriction over the tuning control gain $k_{p}$ to prevent any singularity in the control signal and the use of additional integrators to implement the outer voltage PI loop, the auxiliary dynamics $x_{1🟉}$, and $x_{3🟉}$, and the integral part of the estimator*.

## 4. Real-Time Numerical Results

**Figure 5:**
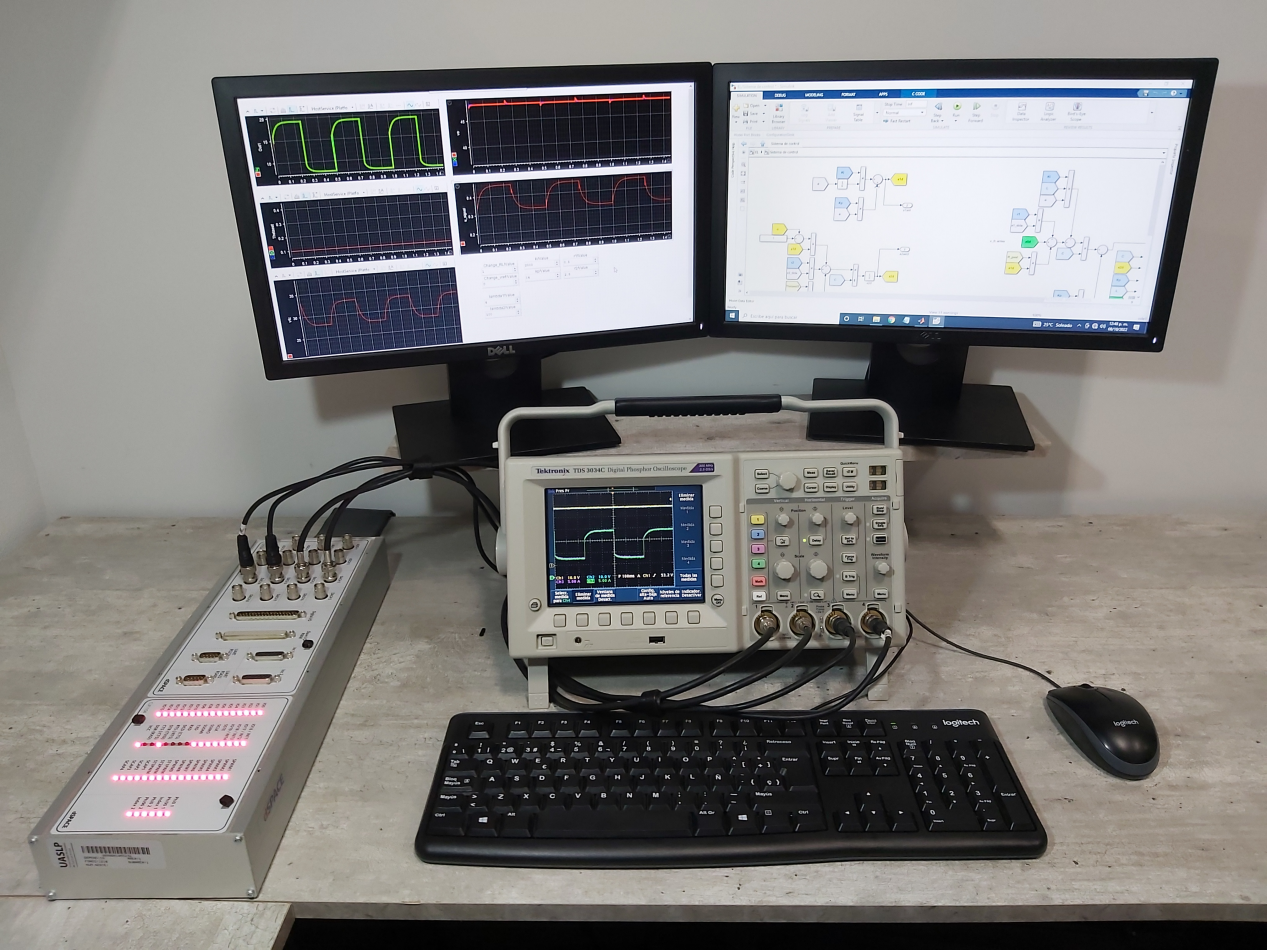
Real-time numerical simulations through DSPACE DS1104, computer, and oscilloscope TDS 3034C.

Real-time simulations are crucial in the industrial sector for operator training, rapid control prototyping, off-line controller tuning, test cost savings, and reducing the design cycle. In this case, the data acquisition DSPACE-DS11004 platform is used (as seen in Figure 5) to run the real-time simulations with the aim to embed the fuel cell/boost converter system (6) in a closed-loop with the proposed adaptive controller stated in Proposition 1. The real-time simulations are configured with the Euler numerical solver and a fixed time step of 50 μs to allow the complete computation of the required adaptive control laws and system expressions. Four independent digital-to-analog (DAC) channels were used to plot the real-time results in a digital oscilloscope to evaluate the closed-loop performance. Recall that the overall control scheme is comprised of: an outer loop based on a PI action over the output voltage error (14); an inner loop designed with E-L-PBC methodology (27), (28), and (30); and ad adaptive law based on I&I (42), as can be seen in Figure 4. The fuel cell system parameters and the adaptive controller gains are given in Table 1. As outlined in the system description, the parameters of the EGS are obtained experimentally. The converter values are chosen to obtain CCM; in addition, the parasitic resistance of the inductor is considered to increase the precision of the model.

For testing the performance of the proposed adaptive controller, two scenarios are considered: sudden stepwise changes in the load and sudden stepwise changes in the output voltage regulation. In the following sections, the testing scenarios are described in detail.

### 4.1. Sudden Load Changes

**Figure 6:**
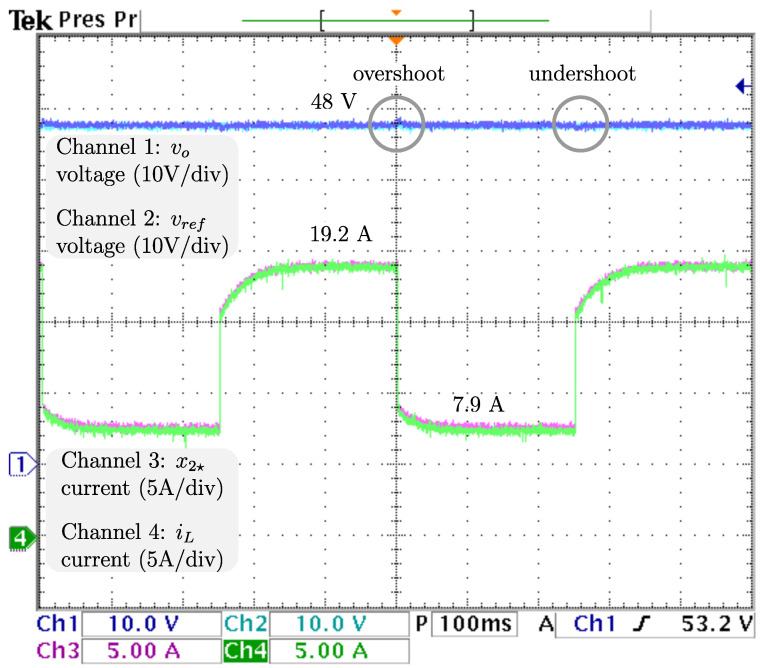
Real -time results for sudden load changes. From top to bottom: $v_{o}$, the output voltage; $v_{ref}$, the reference voltage; $x_{2🟉}$, the inductor current reference; and $i_{L}$, the inductor current.

**Figure 7:**
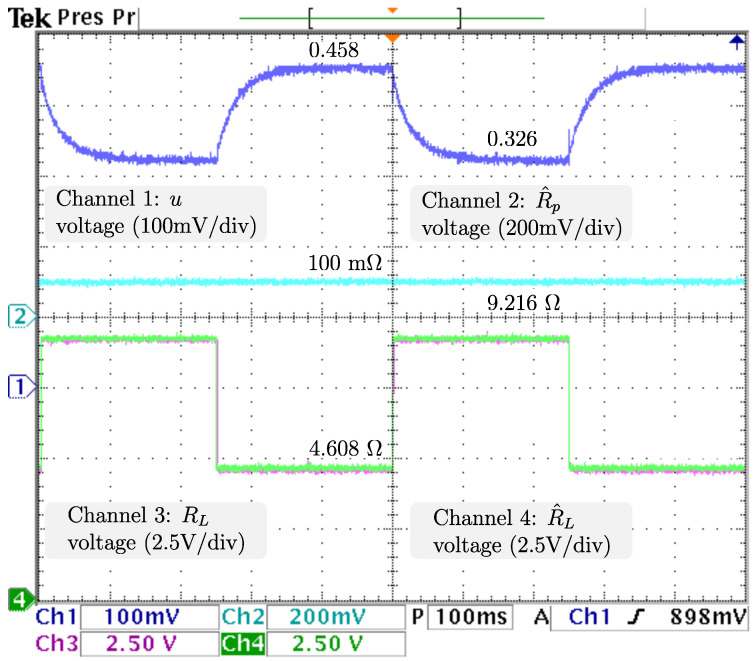
Real -time results for sudden load voltage changes. From top to bottom: *u*, the control signal; ${\hat{R}}_{p}$, the estimation of the parasite resistance; $R_{L}$, the load; and ${\hat{R}}_{L}=1/{\hat{\theta }}_{L}$, the estimation of the load through its conductance.

**Figure 8:**
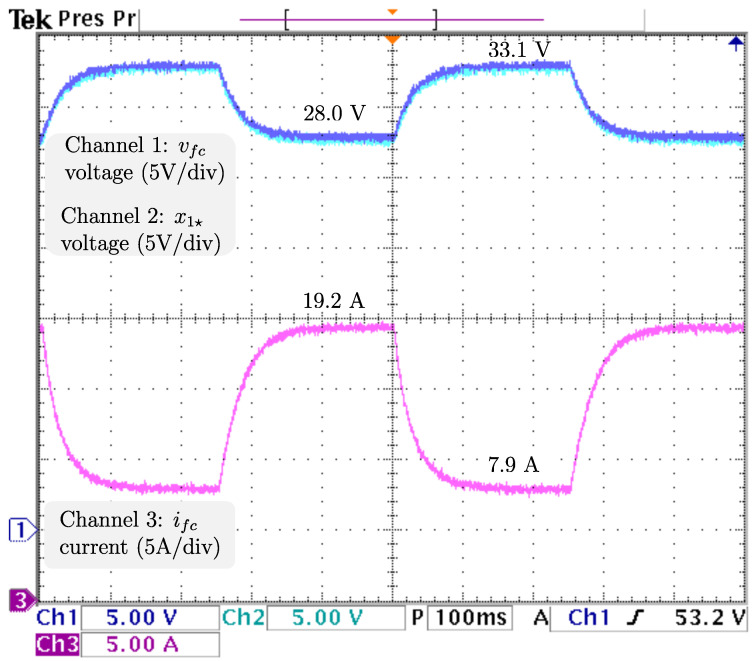
Real -time results for sudden load changes. From top to bottom: $v_{fc}$, the fuel cell voltage; $x_{1🟉}$, the fuel cell voltage reference; and $i_{fc}$, the fuel cell current.

To ensure proper voltage regulation at different loads, $v_{ref}$, the voltage reference is kept at 48 V, and $R_{L}$, the resistive load has sudden stepwise changes at a rate of 5 Hz from the nominal value 4.608 Ω to 9.216 Ω, resulting in an output power drop from 500 W (nominal) to 250 W. The dynamics related to the output voltage and inductor current are displayed in Figure 6 from top to bottom: $v_{o}$, the output voltage; $v_{ref}$, the reference voltage; $x_{2🟉}$, the inductor current reference; and $i_{L}$, the inductor current. It is appreciable that the voltage is tightly regulated, as the difference between $v_{o}$, and $v_{ref}$ is negligible. Additionally, a power drop/surge results in an overshoot/undershoot of less than 0.7 V, acceptable values of less than 2% (0.96 V), with transients of about 100 ms. Hence, as the voltage is tightly regulated (indirectly), proper current reference tracking is achieved. After the power drops $i_{L}$, the inductor current drastically decreases from 19.2 A to more than half the required in less than 5 ms and then decreases smoothly until its demand of 7.9 A is ensured, in about 100 ms. The control signal and adaptive law dynamics are displayed in Figure 7. The signals from top to bottom are *u*, the control signal; ${\hat{R}}_{p}$, the estimation of the parasitic resistance; $R_{L}$, the load; and ${\hat{R}}_{L}=1/{\hat{\theta }}_{L}$, the estimation of the load through its conductance. The closed-loop system response under a power drop is a smooth decrease from *u*, the control law duty cycle from 45.8 % to 36.4 %, with a transient of about 130 ms, and no appreciable undershoot/overshoot. It is fair to say that ${\hat{R}}_{p}$, estimation of the parasite resistance matches the real value at 0.1 Ω, and is tightly regulated at that value. On the other hand, ${\hat{R}}_{L}$, estimation of the load matches the real value, as noticeable when a sudden increase from 4.608 Ω to 9.216 Ω, is tracked in less than 5 ms without overshoots/undershoots. Note that the fast track of the load was designed on the basis of the overall performance of the closed-loop system. The dynamics related to the fuel cell are displayed in Figure 8. The signals from top to bottom are $v_{fc}$, the fuel cell voltage; $x_{1🟉}$, the fuel cell voltage reference; and $i_{fc}$, the fuel cell current. Note that the difference between $v_{fc}$, the fuel cell voltage, and its reference is negligible, thus, the proper voltage reference is achieved. The response after a power drop is a smooth increase in $v_{fc}$, the fuel cell voltage from 28.0 V (nominal) to 33.1 V, and through (7) $i_{fc}$, the fuel cell current decreased smoothly from 19.2 A (nominal) to 7.9 A, both signals with transients about 150 ms, and no noticeably undershoot/overshoot. As outlined in the introduction, this smooth behavior in the fuel cell is desired, because rapid changes in the fuel cell current harm the fuel cell membrane, thus reducing its lifetime. Finally, it is important to mention that the closed-loop system response under a power surge is essentially the same.

### 4.2. Sudden Output Voltage Changes

**Figure 9:**
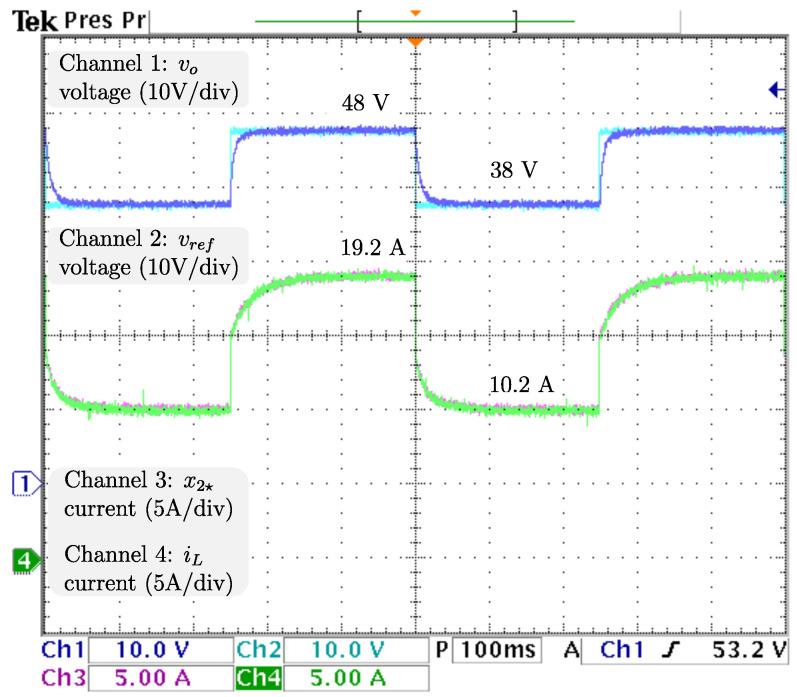
Real-time results for sudden output voltage changes. From top to bottom: $v_{o}$, the output voltage; $v_{ref}$, the reference voltage; $x_{2🟉}$, the inductor current reference; and $i_{L}$, the inductor current.

**Figure 10:**
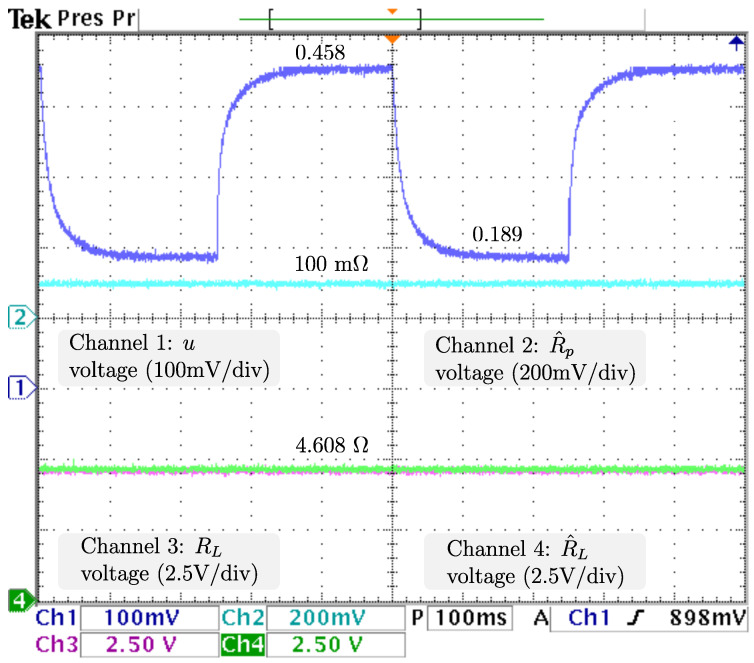
Real-time results for sudden output voltage changes. From top to bottom: *u*, the control signal; ${\hat{R}}_{p}$, the estimation of the parasite resistance; $R_{L}$, the load; and ${\hat{R}}_{L}=1/{\hat{\theta }}_{L}$, the estimation of the load through its conductance.

**Figure 11:**
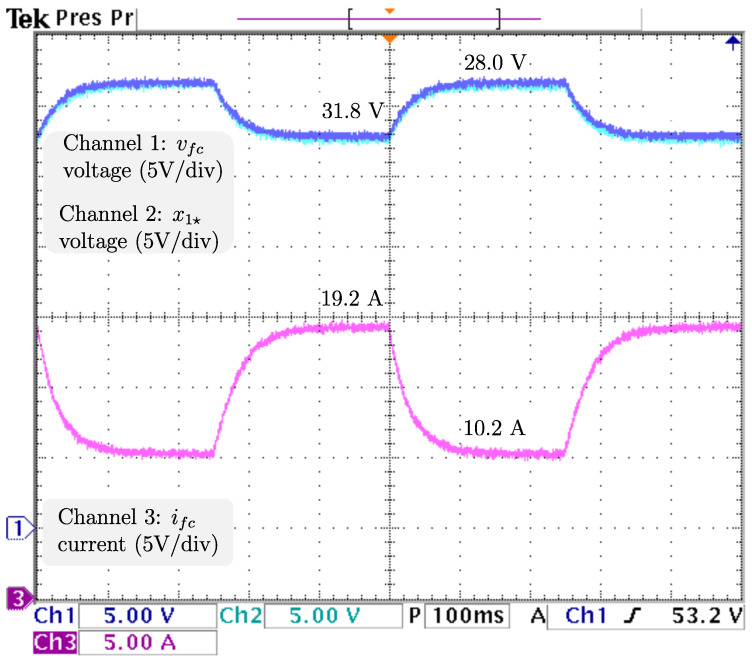
Real-time results for sudden output voltage changes. From top to bottom: $v_{fc}$, the fuel cell voltage; $x_{1🟉}$, the fuel cell voltage reference; and $i_{fc}$, the fuel cell current.

In order to ensure proper voltage regulation at different levels, the load is kept constant at 4.608 Ω, and $v_{ref}$, the voltage reference, has sudden stepwise changes at a rate of 5 Hz from 48 V to 38 V corresponding to an output power of 500 W and 313.368 W, respectively. It is important to mention that power converters generally operate at a constant output voltage level, but it is of interest to evaluate the closed-loop performance when variations in voltage reference are considered. For this scenario, the PI control gains are selected as $k_{p}=0.5$ and $k_{i}=120$. As illustrated in Figure 9, the signals from top to bottom are $v_{o}$, the output voltage; $v_{ref}$, the reference voltage; $x_{2🟉}$, the inductor current reference; and $i_{L}$, the inductor current. The closed-loop system response under a power drop is a tight regulation of $v_{o}$, as changes smoothly to match the reference change from 48 V to 38 V, with transients of less than 50 ms, with no appreciable undershoot/overshoot. Consequently, proper current reference tracking from $i_{L}$ to $x_{2*}$ is achieved. It is noticeable that $i_{L}$ drastically decreases from 19.2 to more than half the required in about 5 ms, and then decreases smoothly until its demand of 7.9 A is ensured, in about 100 ms. The next analysis comprehends the control signal and estimations displayed in Figure 10. Shown from top to bottom are *u*, the control signal; ${\hat{R}}_{p}$, the estimation of the parasite resistance; $R_{L}$, the load; and ${\hat{R}}_{L}=1/{\hat{\theta }}_{L}$, the estimation of the load through its conductance. The closed-loop system response under a power drop is a smooth decrease in *u* from 0.458 to 0.326, with transients of about 130 ms, and no appreciable undershoot/overshoot. Additionally, it is fair to say that estimations of $R_{p}$ and $R_{L}$ remain constant (as should be) at 0.1 Ω and 4.608 Ω, respectively. Thus, their real values are closely estimated. For the last analysis, the dynamics related to the fuel cell are displayed in Figure 11, from top to bottom: $v_{fc}$, the fuel cell voltage; $x_{1🟉}$, the fuel cell voltage reference; and $i_{fc}$, the fuel cell current. The closed-loop system response under a power drop is a smooth increase in $v_{fc}$ to track its voltage reference from 28.0 V (nominal) to 31.8 V, and at the same time, $i_{fc}$, decreases smoothly from 19.2 A to 10.2 A, with transients about 150 ms, and no appreciable undershoot/overshoot.

Because the computational cost of the control is low, the technological feasibility of the proposed controller is assured with a 50 μs times step on the DSPACE 1104 data acquisition platform. Therefore, an experimental setup can be implemented without any issues, and a comparison of experimental results with other control methods can be performed.

## 5. Conclusions

In this paper, an adaptive multi-loop PBC technique for regulating the output voltage of a fuel cell/boost converter system is described and assessed using real-time numerical results. The proposed approach is adaptive because the inductor and load resistances are approximated using an immersion and invariance adaptation law, and consequently the robustness of the overall control scheme is increased by applying these approximations in a certainty-equivalent manner. Furthermore, the inner control loop was developed using standard PBC to ensure accurate tracking of the current reference, which in turn is generated by the outer control loop based on a PI action in order to ensure output voltage regulation. Due to the integration of both loops, the only requirement for implementing the proposed controller is to satisfy a non-conservative constraint on the proportional gain of the outer loop, which is dependent on the physical considerations of the system. Real-time numerical results have demonstrated exact voltage regulation, current tracking, and robust behavior regardless of fuel cell voltage fluctuations, abrupt load, and voltage reference changes. Because the computational burden of the control is low, the technical feasibility of the proposed controller is assured with a 50 μs time step using a DSPACE 1104 data acquisition platform; therefore, an experimental setup can be implemented without any issue. Finally, future research contemplates the following directions: experimental validation of the control laws obtained with a laboratory prototype, and its comparison with other controllers through performance indices. Additionally, hybridization of the fuel cell system with energy storage systems is considered as well, in order to improve the dynamic response.

## Figures and Tables

**Table 1:** Parameters and gains of the adaptive E-L-PBC-based control fuel cell system.

Fuel Cell/Boost Converter Parameters				Control/Adaptative Law Gains			
*a*	2.219	*C*	1.5 mF	$k_{p}$	14	$r_{3}$	2.5
*b*	0.5848	$C_{fc}$	50 mF	$k_{i}$	2500	$\sigma_{1}$	4
$E_{oc}$	40.45	*L*	36.1 μH	$r_{1}$	1	$\sigma_{2}$	100
		$R_{p}$	0.1 Ω	$r_{2}$	0.5		

## Data Availability

The data that support the findings of this study are available from the corresponding author, upon reasonable request.
